# To Talk About It or Not to Talk About It? Social Sharing and the Effects on Psychological and Behavioral Outcomes of Street Harassment

**DOI:** 10.3390/brainsci16020129

**Published:** 2026-01-25

**Authors:** Laura Ferro, Luca Scacchi, Maria Grazia Monaci

**Affiliations:** Department of Social and Human Sciences, University of Valle d’Aosta, 11100 Aosta, Italy; l.scacchi@univda.it (L.S.); m.monaci@univda.it (M.G.M.)

**Keywords:** street harassment, gender violence, social sharing, negative consequences, behavioral changes

## Abstract

**Background.** One of the most common forms of gender-based violence is street harassment, which takes place in public and is usually perpetrated by people who are strangers to the victim. Its diffusion may be a result of its widespread tolerance, and victims often do not protest, denounce, or talk about it with others. **Objectives.** The aim of the present study is to examine social sharing of street harassment episodes between exposure and its mediating effects on negative psychological consequences and behavioral changes for the harassed person. **Methods.** Respondents to an online questionnaire (*N* = 530, 435 F, 8 nonbinary) were asked whether or not they had talked to anyone about their harassment experiences, who they had talked to if they had, and the reasons for not doing so if they had not. **Results.** The results show that one third of our respondents do not report to anyone because the episode was not considered serious, the victim felt ashamed or embarrassed, or believed that nothing would be done; however, this downsizing is associated with increased negative consequences. The relationships between exposure and negative psychological consequences and behavioral changes are partially mediated by the decision not to talk about it. A smaller but still significant mediation shows that the direct relationship between exposure and subsequent behavioral changes is attenuated by talking, while no negative psychological consequences from talking on are observed. **Conclusions.** The implications of these findings suggest that sharing about experiences of harassment can mitigate its negative effects on victims’ quality of life, and people should be encouraged to share and formally report the episodes.

## 1. Introduction

Gender-based violence, often perpetrated in very extreme forms, is a dramatic phenomenon, a significant public health problem, and a serious violation of human rights [1]. One of the most pervasive—despite not being the most serious—form of gender-based violence is street harassment [2,3]. It is characterized by its context, as it is usually perpetrated in public places, and by the strangeness of the relationship between the different actors involved, as it is usually perpetrated by people unknown to the victim [4,5]. The common denominator is the inappropriate and unsolicited display of vulgar and discriminatory attitudes, mainly—but not exclusively—directed at women. Specifically, this includes verbal and nonverbal behaviors such as catcalling, whistling, honking, unwanted appreciation, shouted vulgar or sexually allusive comments, exhibitionism, persistent stalking aimed at unwanted advances, and comments that may be insults regarding ethnicity, religion, social class, and disability, thus demonstrating clear discriminatory intent [6,7,8].

Such acts, performed in an unequal relational context without the victim’s permission, even implicitly, are based on a sexually objectifying and derogatory view of victims [9] and can activate negative feelings and emotions in the object who passively receives these acts, such as embarrassment, disgust, and fear for one’s own safety [10,11,12]. Fredrickson and Roberts [9] proposed a relevant and clear model on the consequences of women experiencing objectification.

They argue that street harassment, like sexual harassment in general, is the result of a sociocultural process of sexual objectification of women, which, by dehumanizing them through a process that Nussbaum [13] specifically calls “negative objectification”, reduces them to parts of their bodies and aligns their value with the eroticized use that others can make of them. The authors’ reflections highlight how “repeated exposure to sexual objectification increases the likelihood that women themselves will then self-objectify” (p. 343).

These phenomena, initially thought to be a peculiar experience of the female sex, is now beginning to be intersectionally observed and studied in other groups, particularly gay and transgender people [4,14,15,16]. Though studies on the experiences of sexual minorities have increased in recent years [15], they are still limited. LGBTQ+ individuals experience higher rates of sexual harassment than their heterosexual counterparts. The number of reports to state or official agencies points to these higher rates of victimization [17]. Street harassment particularly affects individuals whose identities or self-presentations intersect with femininity [15]. This underscores the embedded nature of street harassment as a form of gendered violence. Street harassment is a way in which gender norms, power relations, and sexual objectification are expressed in public spaces.

Research on public space/street harassment suffered by men is even more scarce, despite the growing awareness that men also experience sexual harassment. The few studies that do exist on this topic mainly considered places such as the workplace or the military service [18]; but, to our knowledge, no studies have specifically examined men’s experience of street harassment. A difficulty in this may be that men are less likely to define their own experiences as sexual harassment. Franke [19] expanded the conceptualization of sexual harassment as a means of enforcing rigid gender norms for both men and women. Therefore, the sexual harassment of men serves to punish and control men who have stepped outside of a rigidly prescribed gender role. Men are much more likely to experience harassment from a same-gender perpetrator and in male-dominated environments, where they most commonly experience lewd or vulgar comments or negative remarks enforcing traditional gender role stereotypes [20]. In addition, men are much more likely to be harassed by someone of the same gender as them, while most women are harassed by someone who is a man [18]. These findings all support that street harassment is a way for men to perform their masculinity and assert their dominance over women. A study using the vignette method [21] tested the hypothesis that street harassment may signal dominance. The findings confirmed that male perpetrators who verbally harassed female victims were perceived as dominant with dark personalities. Focusing again on the perceived characteristics of perpetrators, Hindes and Fileborn’s qualitative study [22] strongly supports the impact of gender norms, gender inequality, and hegemonic masculinity on the perpetration of gender-based violence. However, their findings highlight the need to explore more dimensions of power hierarchies and power dynamics that are relational and context-dependent, extending beyond the simplistic claims that street harassment is solely a function of men’s oppression of women.

Cross-cultural research shows that street harassment is common in many parts of the world. More than 50%—and in some studies up to 90%—of women report having experienced at least one episode of harassment in their lifetime, with higher prevalence among younger women and those walking or traveling alone (according to a report by the European Union Agency for Fundamental Rights, 2015, and most surveys, or as reported in online sites such as “Hollaback!”) [23]. The prevalence of street harassment, alongside power dynamics and historically rooted gender status differences [24], may be partly attributable to its widespread perception as a socially tolerated and trivialized form of behavior. Because cultural factors can shape public perceptions of its severity, such behavior is often seen as an integral part of a cultural and social system from which it is difficult to distance oneself by adopting a critical stance.

Despite its prevalence, there are still gaps in the research on this topic. First, there is a lack of a clear definition of street harassment, which sometimes overlaps with ‘sexual harassment’ or ‘catcalling’. As a result, the operationalization of the phenomenon is problematic and validated measurement scales are scarce [25]. Researchers typically propose a list of behaviors and ask people to report whether they have been exposed to them, using dichotomous or frequency scales that refer to different time periods (one month, three years, lifetime, etc.; please see [8] for a review). This heterogeneity also makes it difficult to estimate the prevalence of the phenomenon, which itself varies greatly according to the sociocultural context. Despite the sparsity of existing studies, emerging countries and those with a strong patriarchal component or more widespread machismo and sexism tend to have a higher prevalence of street harassment [25,26] and gender-based violence in general.

One of the problems underlying the uncertain prevalence rate is the lack of formal data on the number of incidents, which is related to the lack of a legal definition of the phenomenon in many countries and the absence of structures dedicated to collecting reports. In addition, victims often do not often protest, ask for a reason, denounce, or report incidents or talk about them with others [27,28], and/or they under-acknowledge their status as victims [29]. The main reasons for this reluctance to report being subjected to street harassment are likely to be its diffuse cultural acceptability, but also the possibility of being singled out as responsible because of the victim’s behavior or clothing; this is especially likely to be the case in cultural contexts strongly dominated by patriarchal patterns, where women fear being defamed or blamed if they report incidents to their family or others [28,30,31].

To date, with the authoritative exception of Fileborn and Vera-Gray’s [31] mixed-methods study and Fileborn’s [32] study on the possibility of sharing harassment incidents on social media, in part to fulfill a need for justice, the research on victims’ reasons for not sharing incidents is scarce.

Trying to understand the reason for the general silence about street harassment and the specific reluctance to report individual incidents also calls into question the aforementioned peculiar legal “hole” around this phenomenon. Street harassment is an unwanted sexual behavior that may or may not involve the physical dimension; together with cyber-bullying, it could harm personal freedom and autonomy, being “intrusive”, whether or not the person’s corporeality is involved. Italian legislation, as in most other countries, focuses on corporeality as a fundamental requirement of crimes against sexual freedom, and does not extend to all cases of non-consensual sexual offenses [33]. Street harassment therefore falls ambiguously both ‘inside’ and ‘outside’ the boundaries of the legal definition of sexual aggression. One of the consequences of this legal gap is a lack of formal authority to report incidents, which, along with widespread cultural acceptance, contributes to the reluctance to talk about them.

The purpose of the present study is to contribute to filling some of the gaps that still exist in the literature by focusing on the reasons why people do or do not talk to others about their exposure to street harassment and, more specifically, by examining the effect of talking or not talking to others on the subsequent negative psychological consequences and behavioral changes and restrictions that victims put in place.

### 1.1. Talking with Others of Street Harassment

Most research and surveys on street harassment have been conducted in university settings. Other recent studies have been conducted in relation to transport systems, particularly in emerging countries, where women, in addition to LGBTQ+ or minority people, are often subjected to harassment [26] (for a review, see [28]). These studies largely confirm the widespread prevalence of these incidents, but once again show that they are rarely reported, not only to official figures, but even to friends and relatives [32]. In Italy [34], the context of our study, a survey among university students confirms the trend found internationally: incidents of harassment have relevant negative psychological consequences (such as anxiety, anger, fear, low self-esteem, self-objectification) and induce behavioral changes; a relevant aspect is that these consequences occur even when the victims do not label the incidents as harassment.

The social acceptance of the phenomenon—although subject to cultural variations and depending on the level of patriarchy and the prevalence of all forms of gender-based violence—means that street harassment is often not seen as violence, but simply as a cultural phenomenon, freedom of speech, relational interaction, or even appreciation [35]; this perception may influence the perceptions held by the victims themselves. A discourse analysis of comments on a posted video of street remarks directed at a young woman [36] indicated that most comments defended the street comments as civil behavior, a form of greeting or compliment; these perceptions outnumbered comments condemning the comments as harassment by a ratio of 2.5:1. The latter highlighted their nature as non-reciprocal, non-intimate, and unwanted interactions, even in the absence of explicitly vulgar or threatening words. In examining public perceptions of street harassment through a vignette-based study, Cullen-Rosenthal and Fileborn [35] emphasized the importance of context in determining whether an incident constitutes harassment; however, many participants set narrow boundaries for what constitutes harmful or acceptable behavior but generally dismiss the cumulative nature of street harassment.

This ambiguity in the perception of the behavior as harassment extends to the victims themselves, especially if they are young women, who are unable to assess the severity of the incident, and this contributes to their silence about what happened. The individual perception of its severity is therefore subjective and influenced by the context [3]. Taking these aspects into account, in our survey, we decided to combine the more common measure of frequency of incidents with a measure of what victims consider to be an actual incident of street harassment; frequency, together with an assessment of the severity of specific harassing behaviors, allows us to obtain a more valid indicator of perceived “exposure” to street harassment.

### 1.2. The Negative Consequences of Street Harassment

In stark contrast to the tendency to trivialize and normalize street harassment, there is evidence that such experiences have a significant negative impact on the lives of those who are harassed, including physical and emotional reactions, severe psychological symptoms, and drastic lifestyle changes [2,25].

Emotional distress may be expressed through feelings of embarrassment, anger, anxiety and/or depression, decreased perceptions of safety, and decreased sleep quality [11,37,38]. Victims often develop high levels of anxiety related to potential physical harm. A qualitative study based on interviews with young high school and university students in Mexico [10] found an increased fear of crime after experiencing street harassment, such as fear of sexual assault, fear of rape, and a generally higher level of insecurity in public spaces.

One of the consequences of exposure to this type of judgment, expressed in the form of catcalling, is the occurrence of self-objectification. This process consists of the internalization of an objectified view of the victim’s own body [39,40]. Women may become intensely concerned with how they appear in public, focus on and constantly monitor their appearance, and experience intense feelings of shame related to their body image. Not knowing the potential aggressor increases risk perception and drastically reduces the victim’s sense of personal safety, significantly increasing the impact and pervasiveness of this negative emotion [12,41]. In addition, the internalization of feelings of invasion, humiliation, and fear associated with harassment experiences has been shown to be a precursor to repressed anger, depression and anxiety, low self-esteem, and relationship problems [42].

### 1.3. Behavioral Consequences and Restrictions

At the behavioral level, the perception of threat and danger can lead women to change their habits or restrict their behavior [4,25,43]. Regarding the strategies used to respond to the experience of harassment, the literature shows that passive strategies are mainly used [44]. It is understandable that victims, who are often alone when faced with one or more harassers, may not react immediately, even in the face of fear of escalating harassment. But even afterward, they often remain passive, reverting to internalized avoidance behaviors to prevent future exposure or to internalized strategies to cope with the negative feelings [10]. According to Magley [45], less than 20 percent of harassed people report or share their experiences with others, while almost all respond with avoidance behaviors: changing routes, choosing a different time of day, avoiding public transportation, avoiding going out alone at night, changing their appearance and posture, revising or restricting their clothing to be less attractive and more “inconspicuous”, avoiding eye contact, or ignoring the street harasser altogether. The combination of these outcomes seems to push victims of street harassment, under the pressure of fear, to find a release from the physical and emotional exhaustion caused by their experience, thus affecting their behavior and socialization patterns and limiting their choices, freedom of action, and movement [43].

The effects of street harassment are thus evident, with immediate physical and psychological manifestations; in the medium–long term, they lead to consequential behavioral changes that significantly reduce the well-being and quality of life of the victims.

### 1.4. Repeated Exposure and the Cumulative Consequences of Street Harassment

One of the consequences of the widespread prevalence and public acceptance of street harassment is that victims are often exposed to repeated episodes over time, although the cumulative effects of such exposure are often underestimated. Street harassment can be included in the category of “non-extreme traumatic experiences”, also called “complex trauma” because of the nature of the victims’ experience and its dysfunctional psychological and behavioral consequences [46,47]. Trauma falls into this category when it is the result of an accumulation of negative life events, chronic unresolved stress, abusive/abusive relationship experiences, or when it is repeated over time, or when it negatively affects a person’s sense of worth, self-confidence, self-esteem, and personal efficacy [47]. Such negative experiences, which would seem to include street harassment, can subjectively lead to intense negative reactions such as sensitivity to emotional activation and reactivity. There is also evidence that these conditions are exacerbated when such traumatic experiences remain unresolved—that is, when the processing of emotions and physical sensations associated with the trauma are impeded [48].

These emotions and bodily sensations linger due to the nature of the unprocessed traumatic experience, creating a neurobiological stasis that activates dysfunctional neural networks that persist beyond the conclusion of the experience, ready to be activated in situations like the traumatic one, even if they are different and safer [49,50]. The literature on traumatic experiences [51,52] has demonstrated the fragmenting effect that such events, especially when repeated and prolonged over time, have on victims’ memories. Repeated traumatic experiences carry the risk of altering memories of facts and their precise place in time, creating dangerous states of confusion in victims, raising doubts about the veracity of events, and leading victims to question their responsibility. By crystallizing the unresolved nature of the trauma, such elements contribute to perpetuating the psychological distress of those who have lived through such experiences [53,54], which also negatively affects their academic, work, relational, and behavioral performance [55].

Research on trauma resolution [51,56] highlights how the narration of traumatic experiences allows for the verbal expression of some of the thoughts and feelings that generate suffering, thus initiating a process of resolution through the construction of new meanings for the experienced suffering [57]. However, victims benefit from talking only when there are conditions (individual and contextual) that allow them to process and integrate the experienced pain into their subjective life experiences [32,58]. What might help explain the reluctance of sexual harassment victims to disclose is an understanding of what happens not only at the individual level, but also at the societal level. In the former case, it is evident that the detailed narrative of the traumatic event re-exposes the victim to the experience of the emotions, cognitions, and physiological and sensory sensations associated with the event. This results in the activation of a negative emotional response in the victim and the defensive response of avoiding the painful reactivation of the memory and the associated psychophysiological response, resulting in the decision not to talk [59]. This reluctance to share can best be understood from the perspective of fear of social judgment or anticipation of negative reactions from others; when sharing takes place in an environment/context that minimizes, denies, reverses responsibility, or offers no solutions for intervention, the most likely outcome is secondary victimization, i.e., the re-traumatization of the victim [60], which increases the risk of psychological vulnerability by activating perceptions of powerlessness, judgment, and guilt. Carretta and Szymanski [11], highlighting the potential negative mental health effects of street harassment on victims, suggest targeting feelings of self-blame, shame, fear, and gender norms in clinical interventions. The pervasiveness of these consequences seems to add a new level of severity to what Davis and Breslau already argued in [61] to be “the invisibility of street harassment and its effects”, and what Fileborn [32] defines as “unspeakable harm”.

### 1.5. The Present Study

The aim of the present study is to investigate the reasons that underlie the decision of whether or not to talk with others about incidents of street harassment by those who have experienced it, and the effects of talking or not talking with others on the consequences of street harassment, taking into account both negative psychological outcomes and behavioral changes undertaken by victims.

The literature suggests a significant relationship between talking with others and the mitigation of negative outcomes from traumatic events [57,62]. Previous studies have primarily used qualitative methods to examine the reasons for not sharing, e.g., [10,25,32], but to our knowledge, our study is among the first to quantitatively examine these reasons and, in particular, their impact on outcomes.

As reviewed, a growing body of evidence suggests that street harassment is associated with negative psychological outcomes [2,12,32,34,38], even when not labeled as such [34,45], and with behavioral changes [7,10,25]. In particular, repeated exposure has negative psychological consequences and induces behavioral changes in an attempt to reduce the effects or the possibility of further episodes.

In the present study, a sufficiently long period of time (three years) was identified during which repeated exposure to such events to induce the cumulative negative consequences of polytraumatic events [11]. In addition, given the possibility of different individual perceptions influenced by the sociocultural context and the specific context of the episode, a subjective rating of the severity of episodes was measured along with their frequency to provide a more reliable index of “exposure” to street harassment. For some victims, catcalling in particular may be considered harmless, if not “just a compliment” or greeting [35,36]. Measuring the prevalence and perceptions of street harassment separately is also one of the final recommendations of a systematic review by Keel and colleagues [8] on operationalizing the construct of street harassment.

Our hypotheses were as follows: First—exposure is significantly and positively associated with outcomes, both psychological and behavioral. Second—exposure is significantly and positively associated with sharing or not sharing episodes of street harassment. Third—there is a significant positive association between sharing and outcomes. Finally, the main hypothesis is as follows: sharing or not sharing serves as a mediator of the relationship between exposure to street harassment and outcomes, including both negative psychological consequences and/or subsequent behavioral changes and restrictions.

## 2. Materials and Methods

### 2.1. Participants

An online survey was conducted in the spring of 2023. The sample was drawn through posts on social networks (e.g., Instagram^®^ and Facebook^®^) and through institutional emails to students at the university in a northwestern region of Italy that conducted the research. The survey was administered online through Google Forms. Participants were required to be 18 years of age or older. With adult participants and anonymous questionnaires, ethical approval was not required according to the national guidelines of the Italian Association of Psychology (AIP). The project was reviewed and approved by the Data Protection Officer of the university where the research was conducted.

A total of 530 respondents (435 identified as women, 86 identified as men, 3 identified as nonbinary, and 6 preferred not identify their gender) constituted our final sample. Regarding sexual orientation, 450 respondents identified as heterosexual, 50 identified as bisexual, 12 identified as gay or lesbian, 6 identified as other, and 12 did not answer this question). Respondent age ranged from 18 to 60 years, with approximately 73% of respondents between the ages of 18 and 24; 361 were students, 142 were employed, 10 were unemployed, and 17 marked “other” as their occupation. Participation in the study was voluntary, and before completing the online questionnaire, respondents read the disclaimer and expressed their consent to participate anonymously and confidentially.

Given the correlational nature of our study, we aimed to collect data on a large sample (i.e., N > 250) that would ensure the stability of the correlations tested and a power of 0.80 for a correlation as low as 0.17, as determined by an a priori power analysis using G*Power (Version N. 3) [63].

### 2.2. Measures

A questionnaire previously used in an Italian university setting [34] was adapted and specifically expanded in the sections on sharing/not sharing harassment episodes with others. The questionnaire was developed using strict gender-neutral language to avoid reinforcing binary or exclusionary gender assumptions.

In addition to the sociodemographic variables (gender with which the respondent identifies, with 4 response alternatives—man, woman, nonbinary/transgender, prefer not to answer; sexual orientation, with 5 response alternatives—heterosexual, gay or lesbian, bisexual, other, prefer not to answer; age group; educational level; occupation), the main variables examined were as follows. The order of the scales and of the items within each scale were fixed.

#### 2.2.1. Exposure to Street Sexual Harassment

Harassment episodes were measured with 13 items describing different types of behaviors and asking how often participants had experienced each behavior in the past three years (in the absence of a validated scale specific to street harassment, the list is based mainly on other items used in previous studies; see, e.g., [2,6]; e.g., “Someone made you feel uncomfortable with a whistling sound”, “Someone started following you”, “Someone touched you without your consent”). The scores for each item ranged from 1 (never experienced) to 5 (experienced very often).

For the same items, respondents were asked to indicate the extent to which each behavior could be considered harassment, again on a 5-point Likert-type scale (from 1 = not at all harassment to 5 = definitely harassment).

The correlation between frequency and severity for each behavior is generally low (ranging from 0.01 to 0.26, with an average correlation of 0.05). An overall indicator was obtained from the relative means for the frequency (α 0.92) and severity (α 0.93) of the reported episodes. In addition, to obtain a global indicator of how the exposed respondents felt to street harassment, the frequency of each harassment incident was weighted by its subjectively perceived severity. For the main analyses, these weighted scores were averaged across the 13 items so that a higher score indicated greater self-reported “exposure” to street harassment (M = 8.09; SD = 3.06, range 1–19.23). The reliability of this overall indicator, as indicated by Cronbach’s alpha, was high (α 0.91).

#### 2.2.2. Negative Psychological Outcomes

To measure the psychological consequences of street harassment, respondents were asked to indicate how often they felt different emotional and psychological states during/after the harassment experience, using 12 items adapted from previous research, e.g., [34,64], and rated on 5-point Likert-type scales (from 1 = never to 5 = always or almost always; e.g., “I became stressed”, “I felt uncomfortable and ashamed about my physical appearance”, or “I had extreme difficulty starting/continuing what I was supposed to do”; these items received the highest mean scores). An overall indicator was calculated from the average of all responses (M = 2.21; SD = 0.94; α 0.92).

#### 2.2.3. Changes in Behavioral Habits

Behavioral changes were measured by asking respondents whether their behavior had been changed as a consequence of the harassment experience with a dichotomous question (yes/no). Following the “yes” response, an ad hoc list of eleven items describing behavioral changes was displayed. Respondents had to select the options that best represented the changes in their lives, allowing for multiple responses. The items were developed based on previous research using interviews and focus groups, e.g., [10,34,65], describing different behaviors (e.g., always telling someone about one’s movements, talking/pretending to talk to someone on the phone, not taking transportation after a certain time; these are the three most commonly reported behaviors). An overall indicator was calculated from the sum of all responses, quantitatively indicating the number of changed behaviors (M = 5.79; SD = 2.45, α 0.92; ω = 0.90).

#### 2.2.4. Sharing

A dichotomous variable (yes/no) examined whether respondents had talked to someone about their harassment experiences. Following a “yes” response (N = 374), a list of items created ad hoc based on previous research, e.g., [30,66], was displayed that indicated six possible individuals with varying degrees of intimacy (parent or guardian, partner, close friend, relative, doctor and/or counselor, law enforcement). Respondents were asked to select the identifiers for the people they had spoken to, allowing for multiple responses. An overall indicator of sharing was calculated from the sum of all responses, quantitatively indicating of the number of people with whom the respondent shared (M = 2.24; SD = 1.01, range 1–5; α 0.71; ω = 0.53).

#### 2.2.5. Reasons Not to Share

In the case of a “no” response to the dichotomous question of whether or not respondents had spoken with someone about their experiences with harassment (N = 156), a list of ten possible reasons was displayed, asking why they had decided not to talk about it. The list was created ad hoc and was partly based on previous surveys mainly conducted at U.S. colleges (Stop Street Harassment, [65]; e.g., “I wanted to forget what happened”; “It is a private matter that I wanted to handle alone”) and qualitative studies, e.g., [10]. Respondents were asked to select the options that best represented their reasons, allowing for multiple responses. An overall indicator was calculated from the sum of all responses, quantitatively indicating the number of reasons not to share (M = 2.07; SD = 1.38, range 1–9; α 0.86; ω = 0.78).

### 2.3. Data Analysis

The data were analyzed using SPSS Statistics for Windows, version 27.0. Preliminary descriptive analyses were conducted on the frequency and severity of harassment, then subgroups by gender and sexual orientation were compared using a Manova followed by post-hoc *t*-tests. The same *t*-test comparisons were also conducted to examine overall exposure to street harassment, behavioral and psychological outcomes, and sharing or reasons for not sharing. Correlations between the study variables were examined. To test the study’s main hypothesis regarding the effects of sharing on behavioral and psychological outcomes, mediation analyses were conducted with a series of regressions based on Baron and Kenny’s [67] guidelines. Three equations were tested separately for the two subsamples of respondents who shared and those who did not share the harassment episodes. First, negative psychological outcomes were regressed on exposure to harassment to establish a mediating effect. Second, the measure of sharing was regressed on exposure to establish the chain of mediation. In the third equation, negative psychological outcomes were regressed on both the exposure and sharing measures. To demonstrate that sharing was functioning as a mediator, the strength of the relationship between the predictor (exposure) and the outcome (negative psychological outcomes) should either be eliminated (a full mediator) or significantly reduced (a partial mediator). Baron and Kenny [67] discussed that it would be unusual, especially in psychological research, for statistical significance to be reduced to zero. Thus, the degree to which the effect was reduced (i.e., the change in the regression coefficients) serves as an indicator of the power of the mediator, and the statistical significance of this reduction in predictive power can be tested using the Sobel test [68] for indirect effects in mediation analysis. The same steps were repeated with behavioral changes as outcome variables.

## 3. Results

Respondents reported that the three most frequent forms of harassment they experienced in the past three years were the following: being looked at in a sexually suggestive way; being whistled at, honked at, or praised unwantedly; being yelled at or called names. The three most severe forms of harassment were the following: someone showing their private parts; being touched without consent (e.g., touching the waist, caressing the breast/chest with the hand, holding the hand, etc.); sexually explicit gestures (e.g., mimicking oral sex, touching the breasts, touching the chest, etc.). Mean values (and standard deviations) are reported in Table 1 in descending order for the total mean values for frequency and perceived severity; these results indicate that the most severe events are likely to be the least frequent.

MANOVAs were conducted on all the scores related to the frequency and perceived severity of the episodes, comparing gender and sexual orientation as independent factors (given the small numbers, participants were grouped as heterosexual, N = 444, 375F, or homosexual/bisexual, N = 68, 49F). The results revealed that the global profiles of respondents who identified as women or men (Wilks’ lambda = 0.57, F_(479,13)_ = 28.5, *p* < 0.001), η_p_^2^ = 0.43) and who identified as heterosexual or homo-/bisexual (Wilks’ lambda = 0.91, F_(486,13)_ = 3.6, *p* < 0.001), η_p_^2^ = 0.087) significantly differed. On the perceived severity judgements, the global profile of respondents who identified as women or men significantly differed (Wilks’ lambda = 0.89, F_(479,13)_ = 4.1, *p* < 0.001), η_p_^2^ = 0ilia.10), while the results were not significantly different between respondents who identified as heterosexual or homo-/bisexual (Wilks’ lambda = 0.96, F_(477,13)_ = 1.5, *p* ns, η_p_^2^ = 0.038).

Examining the appropriate univariates and applying the Bonferroni correction, it emerged (as synthetically reported in Table 1) that harassment episodes were always reported more frequently by women respondents, except for exhibitionism (someone showing their private parts), where the difference was not significant.

A similar trend emerged for the severity scores, with participants who identified as women reporting significantly higher scores, except for two behaviors (“Someone criticized your appearance”; “Someone called you and/or sent you messages of a sexual nature”), where the difference, although always in the direction of higher scores for women respondents, did not reach significance.

Regarding sexual orientation, the differences for the frequency of harassment episodes were almost always significant, with higher frequencies reported by homosexual/bisexual respondents, except for harassment of being followed in the street, which, although in the same direction, was not significant. The situation is different for severity, where the significant differences are far fewer, albeit always in the direction of higher scores for bisexual/homosexual respondents.

Similar results emerged from the *t*-test comparisons of total frequency and severity scores, as well as the weighted indicator of exposure to street harassment, for both gender and sexual orientation (see Table 2). These overall indicators were used in subsequent analyses.

### 3.1. Negative Psychological Outcomes and Behavioral Changes

From the *t*-test comparison by gender, we found that negative psychological outcomes are more relevant for women (M_Females_ = 2.32, DS_Females_ = 0.91 vs. M_Males_ = 1.61, DS_Males_ = 0.82; t (515) = 6.57, *p* < 0.001), and for respondents who self-identify as bi- or homosexual (M_Homo_ = 2.72, DS_Homo_ = 0.89 vs. M_Hetero_ = 2.15, DS_Hetero_ = 0.89; t (506) = 4.43, *p* < 0.001).

Similar results were found for behavioral changes, which are more common in women than in men (M_Females_ = 6.02, DS_Females_ = 2.35 vs. M_Males_ = 3.27, DS_Males_= 2.23; t (363) = 6.05, *p* < 0.001). In contrast, there was no significant difference in behavioral change by sexual orientation.

### 3.2. Sharing Street Harassment

**Figure 1:**
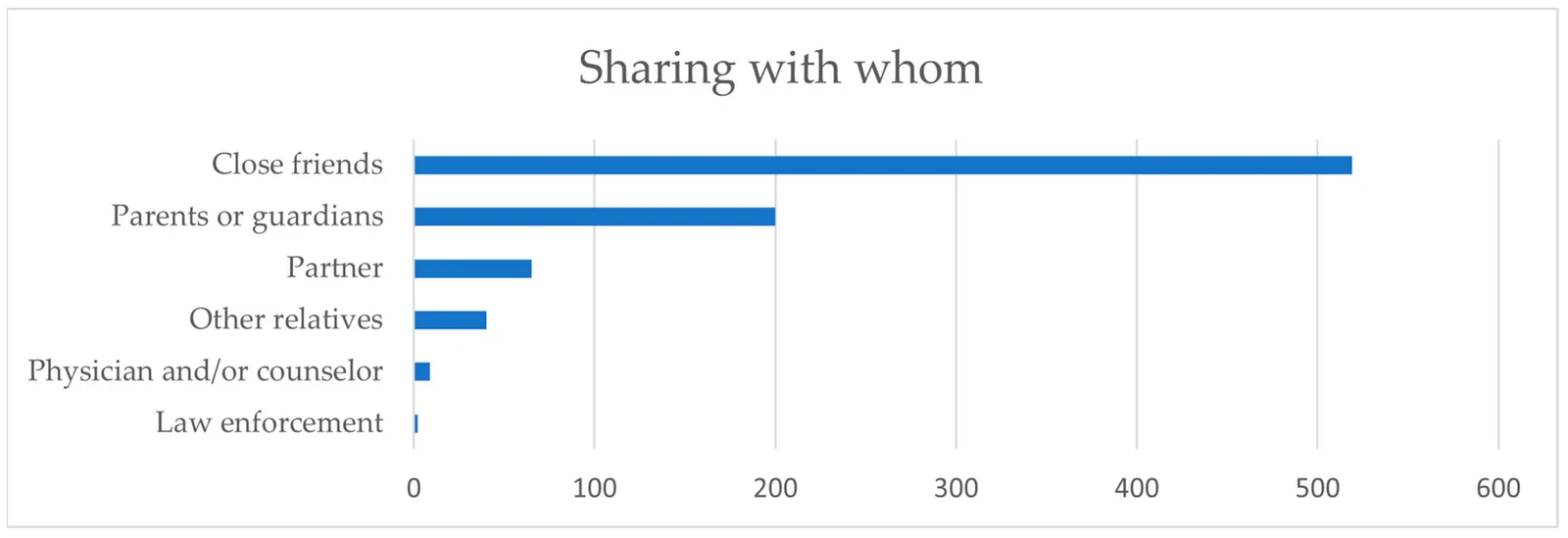
Responses indicating whom the victims chose to share the episodes of street harassment with (N = 373) (question allowed multiple responses).

Figure 1 shows the multiple responses of participants (N = 374; 70.6% of our sample) who reported talking to someone about episodes of street harassment. It shows that almost all reports were made to friends, followed by parents and partners. Virtually no reports were made to counselors or law enforcement.

**Figure 2:**
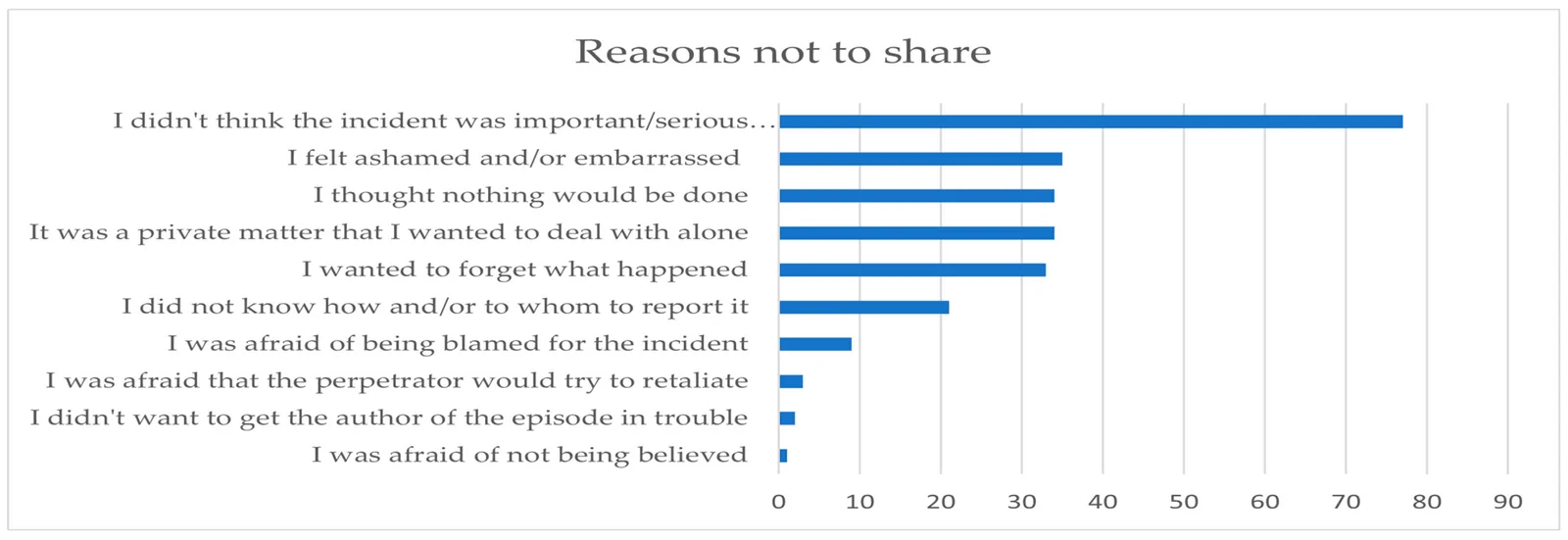
Reasons identified for not sharing the episodes of street harassment (N = 156) by those respondents who chose not to share (question allowed multiple responses).

Figure 2, on the other hand, shows multiple responses regarding the reasons theat people identified for choosing not to talk to anyone about the incidents (N = 156; 29.4%). The most common reasons for not talking about it were that the episode was not considered serious enough, that the victim felt ashamed or embarrassed, that they believed that nothing would be done, or that it was a private matter.

From the *t*-test comparisons for the sum of the total numbers of people telling someone about episodes of street harassment, we found that women were significantly more likely to tell people compared to men (M_Females_ = 2.29, SD_Females_ = 1.01 vs. M_Males_ = 1.86, SD_Males_ = 0.87; t(365) = 2.22, *p* < 0.05). Similarly, women reported significantly more reasons for not talking about it (M_Females_= 2.29, SD_Females_ = 1.01 vs. M_Males_ = 1.86, SD_Males_ = 0.87; t(115) = 2.33, *p* < 0.05). There were no significant differences when sexual orientation was considered.

We then compared the *t*-test results for the two subsamples of people who decided to share or not to share the episodes they experienced with others. Here, the differences were found to be significant when comparing the total score of their frequency and the weighted score of exposure; higher values were identified for the subsample of people who shared with others; meanwhile, a non-significant difference emerged for the total score related to the assessment of the severity of episodes (see Table 1). The subsample of people who shared also reported significantly higher scores for negative psychological consequences and behavioral changes.

Table 3 presents the overall means, standard deviations, and intercorrelations between exposure, frequency, and severity of street harassment, whether the person shared with others or not, and the psychological and behavioral outcomes; these are separated into in the two subsamples of sharing/non-sharing individuals. As expected, there are positive, significant, and moderate/high correlations between exposure to harassment and outcomes, particularly with negative psychological outcomes; meanwhile, correlations with behavioral changes are lower in the sharing subsample. The correlations of the severity ratings with outcomes are instead lower and comparable in both subsamples (although they are significant in the larger sharing subsample).

Also, we found that, the more a person is exposed to these incidents, the more likely they are to talk about them with others; however, this correlation is modest. The correlation between exposure and reasons for not sharing is high. In addition, having many reasons for not sharing is highly and positively correlated with the consequences. On the other hand, the correlations between talking with others and the consequences are modest, though significant (Table 4).

To test for the psychological and behavioral consequences of sharing, four separate mediation analyses were conducted in the two groups of participants who did or did not talk about the harassment episodes; here, exposure to harassment was set as the independent variable and behavioral changes or negative psychological consequences were set as the dependent variables.

Following the steps outlined earlier for testing mediation, we first determined that the predictors were related to the outcome variables. As shown in Table 1, exposure was significantly associated with both negative psychological consequences and behavioral changes, satisfying the condition for mediation in Step 1. Exposure was also significantly associated with sharing and reasons for not sharing, satisfying the condition for Step 2.

To test whether sharing was related to outcome in the first subsample of respondents, we simultaneously regressed negative psychological outcomes on both exposure and sharing (Step 3). This third regression equation also provided an estimate of the indirect path, the relationship between exposure and negative psychological outcomes, controlling for sharing. The direct path was significant (β = 0.57, *p* < 0.001), but the indirect path was still significant (β = 0.55, *p* < 0.001). To assess whether this reduction in the unstandardized regression coefficients (i.e., from the direct path to the indirect path) was statistically significant, the Sobel test estimated the significance of the products of the unstandardized regression coefficients a (between predictor and mediator) and b (between mediator and outcome) and their standard errors. The Sobel test for reduction in indirect mediation between exposure, sharing, and negative psychological outcomes was not significant.

A similar multiple regression was conducted with the other outcome, behavioral changes. The direct path was significant (β = 0.35, *p* < 0.001) and the indirect path was still significant, although smaller than the direct path (β = 0.28, *p* < 0.001), suggesting partial mediation. In this case, the Sobel test was significant (*p* < 0.05).

For the subsample of respondents who preferred not to share the harassment episodes, the direct path between exposure and negative psychological outcomes was significant (β = 0.59, *p* < 0.001). We found regression for the negative psychological outcomes on both exposure and the reason not to share simultaneously, which yielded an indirect path; this was still significant but lower (0.37), and this reduction emerged as significant on the Sobel test (*p* < 0.001). When behavioral change is the dependent variable, the direct path was significant (β = 1, *p* < 0.001), and the indirect path was still significant (β = 0.27, *p* < 0.001), but this reduction emerged as being significant under the Sobel test (*p* < 0.001).

**Figure 3:**
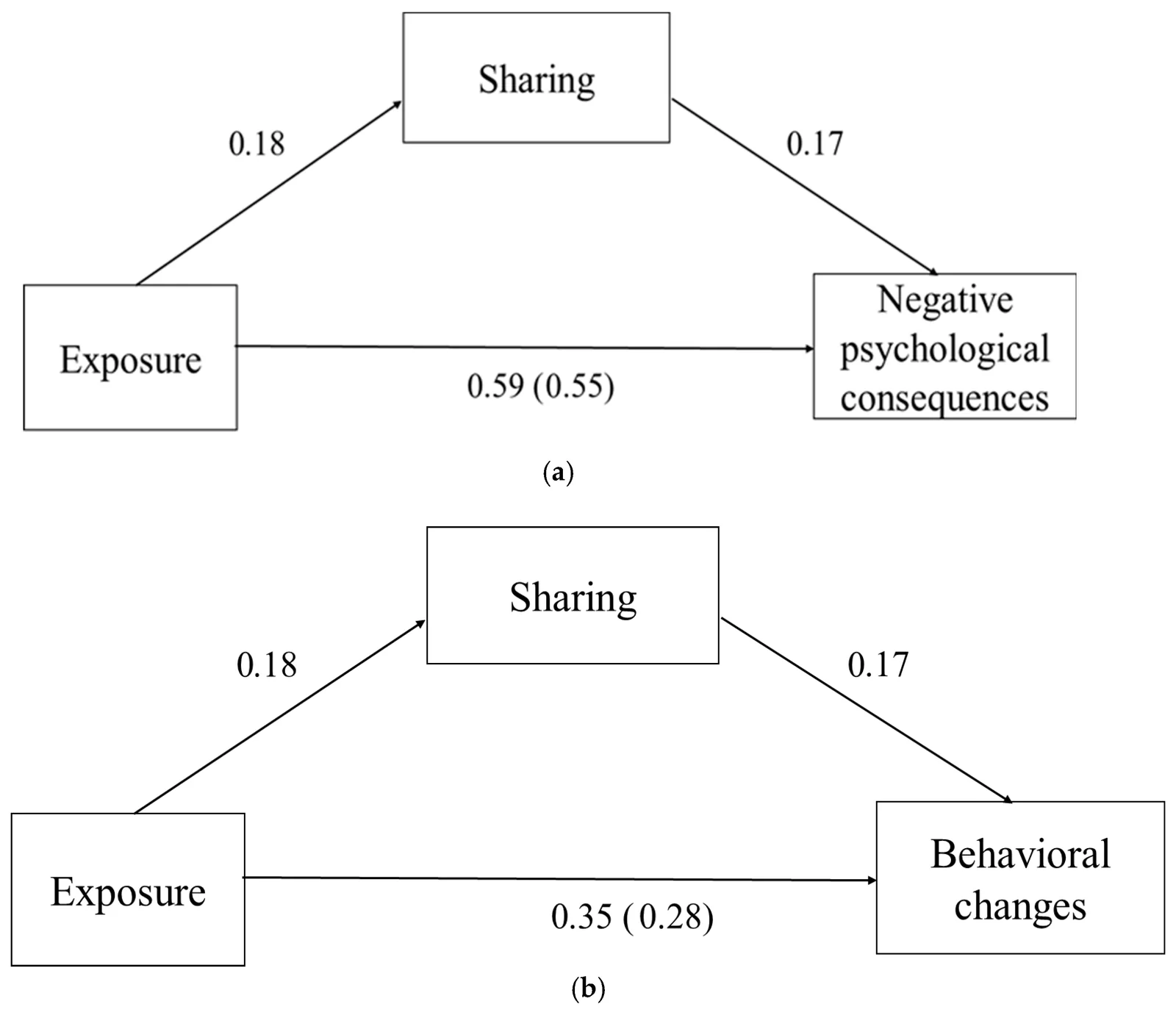
Mediation effects of sharing the details of street harassment episodes with others between exposure to street harassment and negative psychological consequences (**a**) and behavioral changes (**b**). Standardized beta coefficients are reported. The Sobel test for the indirect effect on negative psychological consequences (**a**) does not reach statistical significance, suggesting that there is no mediation effect. The Sobel test for the indirect effect on behavioral changes is significant (*p* = 0.012), suggesting a mediation effect of sharing.

**Figure 4:**
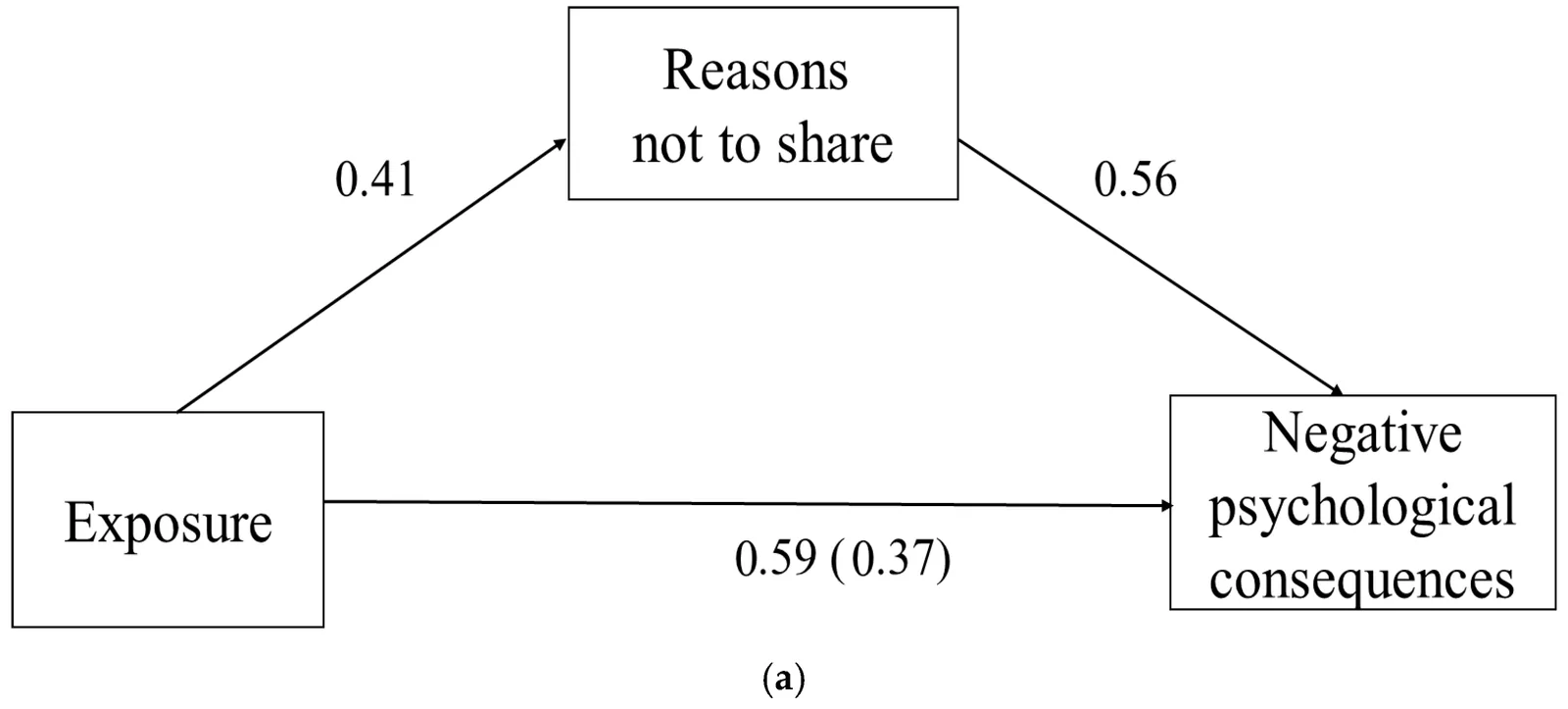

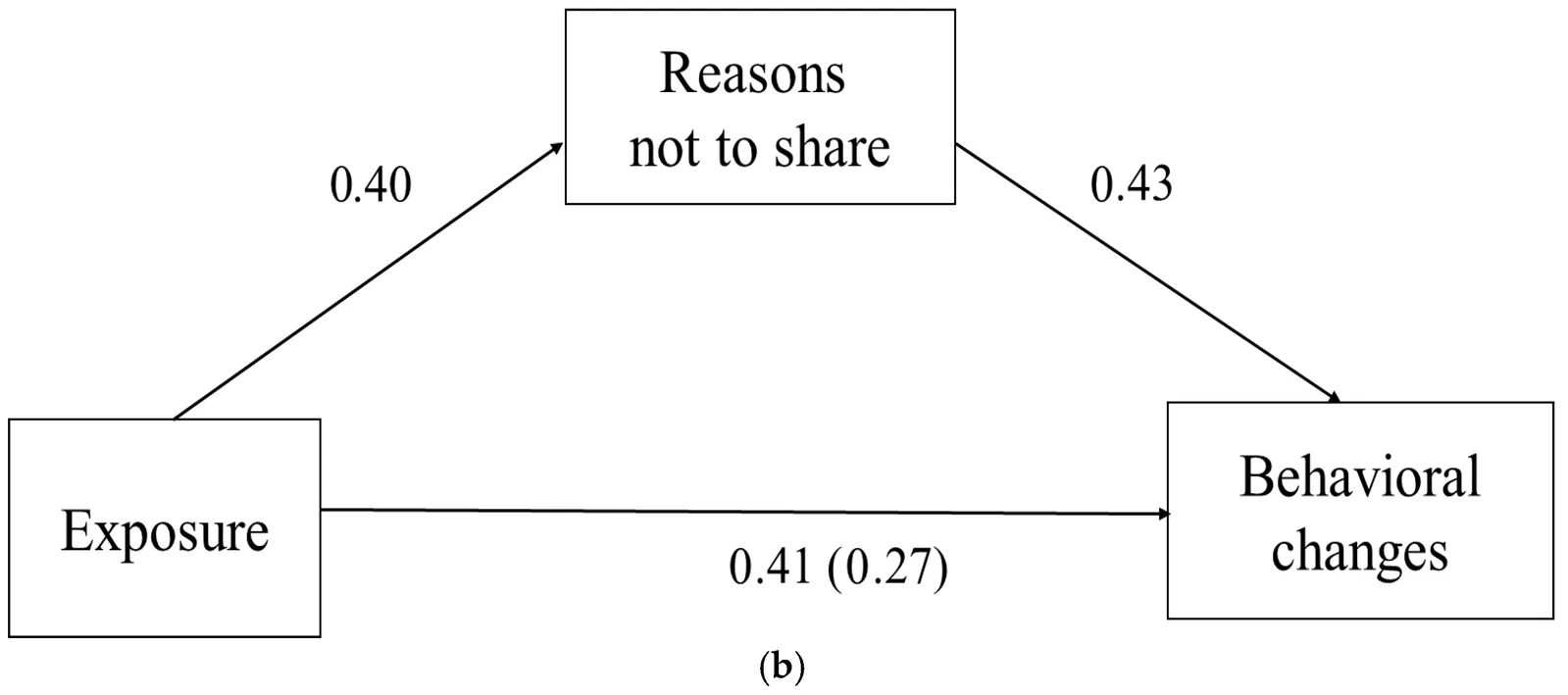
Mediation effects of not sharing between exposure to street harassment and negative psychological consequences (**a**) and behavioral changes (**b**). Standardized beta coefficients are reported. The Sobel test for the indirect effect on negative psychological consequences (**a**) is significant (*p* < 0.001). The Sobel test for the indirect effect on behavioral changes (**b**) is significant, suggesting in both cases a mediation effect of not sharing.

Figure 3 synthetically depicts the results of the mediation tests with negative psychological consequences and behavioral changes as the outcome variables, respectively, in the subsample of respondents who shared the street harassment episodes, where the partial mediation resulted significant in the Sobel test only for behavioral changes. Figure 4 shows the same mediation tests in the subsample of respondents who did not share the episodes, where the Sobel tests were significant for both psychological consequences and behavioral changes.

In summary, sharing is a partial mediator of the relationships between exposure and behavioral changes; meanwhile, having many reasons not to share is a partial mediator of the relationship between exposure and both negative psychological consequences and behavioral changes.

## 4. Discussion

The main aim of the current study was to examine the social sharing of street harassment episodes experienced by victims, identifying whom the victims chose to share with, or, alternatively, the reasons for not sharing. More specifically, the main hypothesis was to test the possible mediating effect of sharing or not sharing on the negative psychological consequences suffered by victims, and the behavioral changes induced by exposure to harassment. To our knowledge, our study is one of the first to specifically address the quantitative impact of sharing or not sharing about experiences of street harassment on a victim, and its negative consequences for them.

Our first finding confirms what has regularly emerged in the literature, e.g., [4,13], that women report more frequent episodes of harassment than men; moreover, we identified that this is also the case for respondents with a non-heterosexual orientation. Similar evidence was found in a US national survey [66], where people who identified as lesbian, gay, bisexual, or transgender were more likely to report experiencing street harassment than people who identified as heterosexual, and these differences were primarily found among men; a systematic review of street harassment surveys found similarly higher prevalence rates among LGBTQI respondents [8]. Due to low numerosity, we could not estimate the mediation effect of sharing on the consequences of street harassment separately for gender identity/diverse sexual orientation. However, in addition to supporting previous evidence on frequency, our findings indicate that people with diverse or non-heterosexual orientations report perceptions of greater severity of street harassment episodes compared to their heterosexual counterparts. They also reported experiencing more severe negative psychological consequences, though their subsequent behaviors and sharing of episodes did not significantly change.

Our results also confirm that exposure to harassment is significantly and strongly associated with both negative psychological consequences and behavioral changes, supporting previous evidence [2,11,25,37,38,43,45]. In the current study, both women and homosexual/bisexual individuals report more severe negative psychological outcomes, while behavioral changes are higher for women but not for homosexual/bisexual persons.

Talking publicly about catcalling or street harassment often results in people being subject to victim-blaming [59,69,70], the mechanism by which people, when confronted with an incident of violence (and especially gender-based violence), place some of the blame for the event on the victim. The results show that most of our respondents (about 70%) talked about the harassment almost exclusively with friends, followed by parents, rarely with their partners, and almost never with law enforcement or authorities. A similar finding was found in a study about sexual harassment on public transport in Lahore [26], where 38% of respondents reported to friends and only 21% reported to family; qualitative group discussions suggested that formal reporting was impossible or avoided for fear of shame or argument. A study of the impact of a campaign to report unwanted sexual behavior on London’s transport system [27] found that only 15% of victims of sexual crime reported incidents to the police, with an increase after the first wave of the campaign. The reasons for not reporting, according to aggregated national police statistics, were “embarrassment”, which was also one of the most relevant in our sample, and people “didn’t think the police could do much to help”.

About a third of our respondents reported that they did not talk to anyone about the harassment they suffered; it is important to analyze their underlying reasons for choosing not to share their experience. We found that, when episodes are not shared with others, it was because they are considered embarrassing or shameful, not particularly serious, or a private and personal matter; another reason for not sharing was that victims felt nothing could be done. It is worth noting that people who reported that they do not share incidents with anyone also reported significantly fewer episodes in the past three years, but their ratings of episode severity did not differ from those reported by the sharing subsample.

However, a lower frequency of exposure—operationalized in this study as a weighting of frequency with the severity of episodes—alone does not seem to be one of the main motivations for not sharing. The reasons for not sharing reported by these individuals all point to a reduction in the salience of street harassment and the adoption of passive avoidance strategies. However, at the same time, the number of reasons given for not sharing is strongly associated with both exposure on the one hand and an increase in negative outcomes on the other. Thus, the more one is exposed to street harassment, the more reasons one has not to share, and the more negative the consequences.

In contrast, exposure to harassment is less correlated with the number of people to whom the incidents are reported, and sharing itself is also modestly directly related to negative consequences.

Testing our main hypothesis—that sharing or not sharing with others mediates the relationships between exposure and negative outcomes of street harassment—it emerged that sharing was a partial mediator of the relationship between exposure to street harassment and subsequent behavioral changes (such as always telling someone about one’s movements; talking/pretending to talk to someone on the phone; not taking transportation beyond a certain time) in the subsample of respondents who chose to talk about street harassment. Here, there was a significant, albeit modest, reduction in the magnitude of the relationship between exposure and behavioral change. On the contrary, sharing was not a mediator of the relationship between exposure and subsequent negative psychological consequences (e.g., feeling stressed, feeling discomfort and shame about one’s appearance, having extreme difficulty starting or continuing to do what one is supposed to do). Therefore, the more victims talk about their experience, the more the direct relationship between being harassed and subsequent changes in behavior is attenuated; meanwhile, no reduction in negative psychological consequences is observed.

In contrast, in the subsample of people who choose not to share the episodes, both the relationships with the behavioral and psychological negative consequences of harassment are partially mediated by having many reasons not to talk to others about harassment episodes. In this case, the direct relationships between exposure and both negative psychological consequences and behavioral changes are significantly reduced by having many reasons not to share.

The episodes of street harassment can be ambiguously interpreted as aggression or not, and the subjectivity of this interpretation, together with the public perception and widespread acceptance of it, can lead to the victim not recognizing that they are a victim. A qualitative study with an online survey and focus groups that specifically explored participants’ reasons for and experiences with disclosing their encounters with street harassment suggested that victims want their experiences to be taken seriously and listened to by others [32].

We can conclude that the widespread social acceptance of phenomena such as catcalling and the tendency to blame the victims of gender-based violence have a clear influence on victims, who downsize the importance of these incidents and are reluctant to report them in the face of a feeling of helplessness. The findings of our study add to the existing evidence that highlights that this downsizing is associated with increased negative consequences; at the same time, it contributes to partially reducing the direct relationship between exposure and the same negative consequences, at least in the medium term.

However, especially in cases where people experience repeated episodes, this avoidant coping [60] may lead to the typical consequences of posttraumatic stress disorder [49,58,70] by crystallizing the non-resolution of the trauma and maintaining the psychological distress of those who have lived through such experiences [53,54]. Evidence on the negative consequences of traumatic events suggests a significant relationship between talking with others and their mitigation [52,62]. Specifically for street harassment, as Fileborn [32] remarks, disclosure is essential to making its harms visible and working to change social and cultural attitudes. As Carretta and Szymanski [11] suggest, women and victims in general should be encouraged to engage in activism, which can help prevent self-blame and shame. In addition to the possible resulting empowerment of women and minority groups, activism can be directed towards achieving social justice [31,32].

Victims are thus doubly victimized: by the street harassment episodes themselves and by the social pressure that leads them to adopt an attitude of trivializing, forgetting, and ignoring the episodes. It is important to emphasize that, based on our results, when victims decide to talk about these incidents with others, it at least leads to a mitigation of subsequent behavioral changes with an improvement in the victims’ quality of life.

### 4.1. Limitations and Future Research Directions

This study has limitations. One of these is inherent in the nature of any online survey and the relative sampling process, which results in an unrepresentative sample of the population exposed to harassment. Our sample is limited in the number of men and people who identify as nonbinary, as well as in the number of people who report a non-heterosexual orientation. At the same time, these are the subgroups that the literature and our own findings suggest are most likely to experience street harassment, along with women. Therefore, the generalizability of our findings may be limited to heterosexual women, while future studies could focus on subpopulations of different gender identities and incorporate nonbinary and intersectional perspectives into research on street harassment [15,22]. Another limitation is the lack of validated questionnaires about the sharing of harassment; the present study and its findings constitute a step in this direction.

The cross-sectional nature of the study also hinders the possibility of causal explanations. Our mediational analyses highlight the possible effects of sharing and, in particular, of not sharing on negative outcomes. Although the reasons for not sharing partially mediate the relationship between exposure and outcomes, with a reduction in negative psychological and behavioral consequences, at least in the medium term, our study does not allow us to determine whether the long-term consequences of repeated exposure that may lead to PTSD are present or even more relevant in people who did not share their episodes. Future longitudinal studies would support a better understanding of the underlying mechanisms.

Despite its limitations, the present study offers at least two original and innovative contributions. First, it highlights the opportunity to consider both the frequency and perceived severity of street harassment episodes contemporaneously, which is especially important because, often, the victims themselves do not recognize or admit that such episodes constitute a form of gender violence. Second, it is a first attempt at quantitatively investigating the understudied phenomenon of sharing street harassment experiences. The decision of whether or not to share them may impact the negative consequences of this widespread gender violence, and this should be considered in prevention interventions.

### 4.2. Practical Implications

The implications of our findings lead us to consider how creating conditions that facilitate the sharing of harassment could reduce its negative impact on victims. People who experience street harassment may benefit from reporting it; this seems to be especially the case for those who do not feel compelled to change their behavior as a result of the harassment. So, the focus should be on the difficulties victims have in sharing the episodes with others and the reasons they have for not doing so. In addition, although most of our respondents talked to someone, they almost never publicly or formally reported the episodes to law enforcement, counselors, or support professionals.

Cultural differences and sociopolitical practices affect attitudes toward this type of gender-based violence and the varying likelihood that victims will recognize and report harassment as such. In more aware contexts, victims may receive social support that facilitates identification and formal reporting. In Italy, as in other European and non-European countries, street harassment is not yet considered a crime [33] and therefore there are no specific penalties for it, but depending on the specific case, it can constitute two different crimes: stalking or “harassment or annoyance of persons”. In the latter case, it is an offence that punishes whoever, in a public place or a place open to the public, or by means of the telephone, for petulance or any other blameworthy motive, harasses or annoys someone. What abstractly distinguishes catcalling from the crime of harassment or annoyance is the legal right protected by the norm: traditionally, it is believed that this crime is intended to punish the disturbance of public tranquility, not the dignity of the offended person being harassed. The absence of specific regulations against this type of conduct may therefore be one of the reasons for its trivialization, since there is no “crime” for which a harasser can be reported. This lack of institutional response increases the possibility of secondary victimization: the victims, confused by the helpless reaction of the context, come to formulate self-denigrating and destructive thoughts, identifying themselves as responsible, experiencing negative emotions of anxiety and fear, combined with feelings of guilt that should instead belong to the harasser [71]. The worst element is that victims, discouraged from speaking openly about the distressing situation, give up on asserting their rights. This resulting secondary victimization is often doubly linked to gender prejudices and stereotypes.

Public activism can play an important role against incidents of street harassment. In fact, much information and mobilization are effectively carried out through blogs and social channels [32], as acting in community and confronting one’s experiences generates effects of recognition and support and can play a function of informal justice. Recently, collectives promoted by women university students on Twitter and Instagram (#ChalkWalk; @HeartMob) have invited the sharing of messages denouncing offensive approaches and have encouraged making them public. For instance, writing them in colored chalk, in block letters, and in the places where they occurred. This is a useful and courageous experiment that also configures a successful tool for the strategic therapy of posttraumatic stress disorder: the externalization of memories, images, and flashbacks, and the reclaiming of a physical space that has tended to be avoided because it is associated with unpleasant moments, can increase a person’s confidence in their resources.

## 5. Conclusions

The negative consequences of street harassment are often overlooked, and recognizing its explicit nature as gender-based violence is an important societal task. Previous evidence [2,37,38,43,45] and our findings show that victims are generally severely affected by the behavioral and psychological consequences of street harassment.

Street harassment is an “unspeakable” harm that is usually silenced, and victims’ experiences are trivialized [32]. Our study is one of the few to examine the effects of sharing or not sharing episodes of street harassment on negative behavioral and psychological outcomes. Based on our findings, the decision to report to others at least attenuates subsequent behavioral changes. On the other hand, one third of the victims reported that they do not share their experience of street harassment with anyone. This decision seems to be made in a logic of ignoring the episodes as much as possible because they are perceived as not being serious, being private, or being embarrassing, or because the victim is under the impression that nothing would be done to help them. This downsizing of street harassment, which induces people not to talk about the incidents, is associated with increased negative consequences. Some of our results indicate that it may have positive consequences, reducing both the psychological and behavioral consequences of the exposure, at least in the short term.

We can conclude that more education about the feelings and experiences of victims of street harassment could significantly reduce dysfunctional communication, confusion, and the downsizing and passive strategies often adopted by victims. Opportunities should be created where victims of street harassment can feel welcome and talk about the violence they have experienced, especially in formal contexts and with authority figures, to help create a social context that no longer considers these forms of gender-based violence to be normal and acceptable.

## Figures and Tables

**Table 1:** Mean values of frequency and perceived severity of street harassment episodes.

	Mean SD				Mean SD		
Frequency of street harassment				Severity of street harassment			
Someone looked at you in a suggestive, sexual way.	2.8	1.1		Someone showed you their private parts.	4.8	0.7	o
Someone made you feel uncomfortable by whistling, honking, or making unwanted comments.	2.8	1.1		Someone touched you without your consent.	4.8	0.7	o
Someone shouted obscenities at you.	2.3	1.0		Someone made sexually explicit gestures (e.g., mimed oral sex, touched their private parts, etc.).	4.7	0.7	o
Someone criticizing your appearance.	2.2	1.0		Someone asked you to perform sexually explicit acts or offered you money in exchange for sex.	4.7	0.9	o
Someone touched you without your consent.	2.0	1.0		Someone started following you.	4.6	0.8	
Someone slowing down or stopping their car to get closer to you.	2.0	1.0	o	Someone has made calls and/or sent messages of a sexual nature to you.	4.5	0.9	g o
Someone has repeatedly attempted to arrange an unwanted meeting with you.	1.8	1.0		Someone shouted obscenities at you.	4.3	1.0	o
Someone has made calls and/or sent messages of a sexual nature to you.	1.8	1.0		Someone has repeatedly attempted to arrange an unwanted meeting with you.	4.2	1.1	
Someone started following you.	1.7	0.9	o	Someone slowing down or stopping their car to get closer to you.	4.2	1.0	
Someone has repeatedly attempted to arrange an unwanted meeting with you.	1.6	0.9		Someone looked at you in a suggestive, sexual way.	4.1	1.1	o
Someone made sexually explicit gestures (e.g., mimed oral sex, touched their private parts, etc.).	1.6	0.9		Someone made you feel uncomfortable by whistling, honking, or making unwanted comments.	4.0	1.2	
Someone asked you to perform sexually explicit acts or offered you money in exchange for sex.	1.3	0.7		Someone has repeatedly attempted to arrange an unwanted meeting with you.	3.9	1.2	
Someone showed you their private parts.	1.3	0.7	g	Someone criticizing your appearance.	3.7	1.2	g

Note. The mean values are reported in descending order for the total mean values, respectively, for frequency and perceived severity. In the third column, non-significant values are reported for the *t*-test comparisons: g indicates a non-significant difference according to gender; o indicates a non-significant difference according to sexual orientation.

**Table 2:** Mean values and *t*-test comparisons for gender and sexual orientation on overall frequency, perceived severity, and exposure.

	Total Mean (SD)		Mean F		Mean M		*t*-Test	Hetero		Homo/Bisex		*t*-Test
	**(N = 530)**		**(N = 435)**		**(N = 86)**			**(N = 450)**		**(N = 68)**		
Overall frequency	25.2	7.8	26.6	7.5	18.2	5.5	<0.001	24.6	7.5	28.9	8.8	<0.001
Overall severity	4.3	0.7	4.4	0.6	4.0	1.0	<0.001	4.3	0.7	4.5	0.6	0.029
Exposure	8.2	3.1	8.7	3.0	5.7	2.2	<0.001	8.0	2.9	9.8	3.4	<0.001

Note. Nonbinary individuals and those who preferred not to answer were excluded from the analyses.

**Table 3:** Means, standard deviations, and *t*-test between sharing/non-sharing subsamples for the study variables.

	Sharing N = 373		Not Sharing N = 153		
	**Mean**	**SD**	**Mean**	**SD**	***p***
Frequency	26.95	7.56	20.94	6.85	0.001
Severity	4.36	0.76	4.27	0.71	ns
Exposure	8.88	3.03	6.78	2.65	0.001
Negative psychological consequences	2.31	0.91	1.97	0.96	0.001
Behavioral changes	6.06	2.43	4.81	2.31	0.001

**Table 4:** Means, standard deviations, and correlations among the study variables in the sharing/non-sharing subsamples.

	MEAN	SD	1	2	3	4	5	6	7
1. Exposure ^b^	8.19	3.06	─	**0.88**	**0.49**	^a^	**0.40**	**0.57**	**0.41**
2. Frequency ^c^	25.2	7.85 ^a^	**0.88**	─	0.09	^a^	**0.36**	**0.58**	**0.45**
3. Severity	4.33	0.72	**0.56**	**0.17**	─	^a^	0.20	0.17	0.20
4. Spoken ^c^	2.24	1.00	**0.18**	**0.19**	0.05	─	^a^	^a^	^a^
5. Reasons not to talk about it ^c^	2.07	1.38	^a^	^a^	^a^	^a^	─	**0.57**	**0.44**
6. Negative psychological outcomes ^c^	2.21	0.94	**0.57**	**0.55**	**0.27**	**0.20**	^a^	─	**0.48**
7. Behavioral changes ^c^	5.79	2.45	**0.30**	**0.26**	**0.24**	**0.17**	^a^	**0.32**	─

Note. Correlations above the diagonal are for the non-sharing subsample; correlations below the diagonal are for the sharing subsample. Correlations in **bold** are significant at *p* < 0.005. ^a^. Impossible to compute because of the two different subsamples of respondents. ^b^. Ponderation between frequency and severity. ^c^. Sum of the multiple responses.

## Data Availability

The data that support the findings of this study are available from the corresponding author upon reasonable request. Since these data are protected under the university’s data protection regulations, they cannot be permanently exported from the university’s IT platforms except upon a specific and justified request; in all cases, the anonymity of the respondents and the non-identifiability of the individuals involved must be ensured.
